# Mapping EQ-5D utilities to GBD 2010 and GBD 2013 disability weights: results of two pilot studies in Belgium

**DOI:** 10.1186/s13690-017-0174-z

**Published:** 2017-02-06

**Authors:** C. Maertens de Noordhout, B. Devleesschauwer, L. Gielens, M. H. D. Plasmans, J. A. Haagsma, N. Speybroeck

**Affiliations:** 1 Institute of Health and Society (IRSS), Université catholique de Louvain Clos Chapelle-aux-Champs, 30 bte B1.30.15, Brussels, 1200 Belgium; 2 Department of Public Health and Surveillance, Scientific Institute of Public Health, Rue Juliette Wytsman 14, 1050 Brussels, Belgium; 3 National Institute for Public Health and the Environment, Centre for Health and Society, P.O. Box 1, 3720, BA Bilthoven, The Netherlands; 4 Department of Public Health, Erasmus MC, Dr. Molewaterplein 50, 3015 GE Rotterdam, The Netherlands; 5 Institute for Health Metrics and Evaluation, University of Washington, Seattle, WA 98121 USA

**Keywords:** EQ-5D, Utilities, GBD2010, GBD2013, DW, Mapping

## Abstract

**Background:**

Utilities and disability weights (DWs) are metrics used for calculating Quality-Adjusted Life Years and Disability-Adjusted Life Years (DALYs), respectively. Utilities can be obtained with multi-attribute instruments such as the EuroQol 5 dimensions questionnaire (EQ-5D). In 2010 and 2013, Salomon et al. proposed a set of DWs for 220 and 183 health states, respectively. The objective of this study is to develop an approach for mapping EQ-5D utilities to existing GBD 2010 and GBD 2013 DWs, allowing to predict new GBD 2010/2013 DWs based on EQ-5D utilities.

**Methods:**

We conducted two pilot studies including respectively four and twenty-seven health states selected from the 220 DWs of the GBD 2010 study. In the first study, each participant evaluated four health conditions using the standard written EQ-5D-5 L questionnaire. In the second study, each participant evaluated four health conditions randomly selected among the twenty-seven health states using a previously developed web-based EQ-5D-5 L questionnaire. The EQ-5D responses were translated into utilities using the model developed by Cleemput et al. A loess regression allowed to map EQ-5D utilities to logit transformed DWs.

**Results:**

Overall, 81 and 393 respondents completed the first and the second survey, respectively. In the first study, a monotonic relationship between derived utilities and predicted GBD 2010/2013 DWs was observed, but not in the second study. There were some important differences in ranking of health states based on utilities versus GBD 2010/2013 DWs. The participants of the current study attributed a relatively higher severity level to musculoskeletal disorders such as ‘Amputation of both legs’ and a relatively lower severity level to non-functional disorders such as ‘Headache migraine’ compared to the participants of the GBD 2010/2013 studies.

**Conclusion:**

This study suggests the possibility to translate any utility derived from EQ-5D scores into a DW, but also highlights important caveats. We observed a satisfactory result of this methodology when utilities were derived from a population of public health students, a written questionnaire and a small number of health states in the presence of a study leader. However the results were unsatisfactory when utilities were derived from a sample of the general population, using a web-based questionnaire. We recommend to repeat the study in a larger and more diverse sample to obtain a more representative distribution of educational level and age.

**Electronic supplementary material:**

The online version of this article (doi:10.1186/s13690-017-0174-z) contains supplementary material, which is available to authorized users.

## Background

Disability weights (DWs) and utilities (also known as index values, preference weights or QALY weights) are common metrics allowing a quantitative evaluation of health states related to diseases, injuries or risk factors. They reflect the preference of individuals for a certain health state and quantify the severity of health loss associated with a certain health state. They are also crucial components in health economic evaluations and in disease burden assessments.

Disease burden assessments using the Disability-Adjusted Life Year (DALY) metric play an increasingly important role in public health research. The disability weight (DW) is a crucial component in the DALY formula, as it allows combining morbidity and mortality [1–4, 5, 6]. The DW expresses the relative reduction in Health-Related Quality of Life (HRQoL), on a scale from zero (perfect health) to one (worst possible health state). By weighing the years lived with disease, these years are “translated” into fully lost life years. Indeed, living 10 years with a 10% reduction in HRQoL (i.e., DW = 0.10) would be equal to losing one full year of good health (e.g., by dying one year before the life expectancy). As a result, both morbidity and mortality are expressed in the same currency, namely the number of healthy life years lost. Initially, DWs were elicited from participants who did not necessarily suffer from the studied health state, using time trade-off or person trade-off methods. However other valuation techniques for eliciting DWs exist [7]. Ideally DWs should be developed from international studies hence ensuring internal comparability. However, several health states are not included in such international initiatives. Therefore resorting to specific elicitation studies is the only option. For instance, to estimate the YLDs of stage 0 (in situ) melanoma in Belgium during the two first month of treatment, Tromme et al. multiplied the number of patients with stage 0 melanoma in Belgium for the 2009–2011 period, i.e. 1772 patients by the derived DW for the two first month of treatment, i.e. 0.3345 and by the duration, i.e. 2 months [8] but the DWs obtained in these standalone studies are not comparable to an international set of disability weights.

As part of the Global Burden of Disease 2010 Study (GBD 2010), Salomon et al. proposed a wide set of DWs based on paired comparison questions for 220 health states in which respondents considered two health outcomes which were described briefly in lay language [1]. An update of these DWs was generated for the Global Burden of Disease 2013 Study (GBD 2013) by incorporating results of new surveys in four European countries (Hungary, Italy, the Netherlands, and Sweden). The two studies combined resulted in a set of DWs based on 60,890 participants [2, 3].

In health economic evaluations, health “utilities” are a crucial component of Quality-Adjusted Life Years (QALYs). A utility of 0 means that a health state is equivalent to death, whereas a utility of 1 means full health. These values can be derived using multi-attribute instruments such as the EuroQol 5 dimensions questionnaire (EQ-5D) [9]. The EQ-5D includes five dimensions (mobility, self-care, usual activities, pain/discomfort and anxiety/depression) and five severity levels in each dimension (EQ-5D-5 L). This self-completion questionnaire was developed by the EuroQol group and is applicable to a wide range of patient’s health states.

To derive utilities, individuals are asked to score their health state using EQ5D questionnaire. Their scores may then be converted to a utility using a set of regression coefficients (or tariffs). These tariffs are obtained using direct valuations of a subset of health states from the general public using visual analog scale or trade-off methods and are available for certain countries. Some countries have integrated EQ-5D in their national health surveys, resulting in population average utilities that can be used as an indicator of general population health [10–14].

Many studies calculated utilities using EQ-5D for a wide range of diseases such as cancer [8], cardiovascular diseases [15] or diabetes [16]. EQ-5D has also been applied in the context of DALY-based disease burden studies. In these instances, the EQ-5D utilities had to be “translated” to DWs. So far, authors have assumed the DW to be equal to one minus the utility, or equal to the age and sex specific population average utility minus the elicited utility [8, 17]. However, these approaches do not guarantee comparability with the GBD 2010/2013 DWs. Mapping is a better strategy to predict DWs from utilities when DWs are not available for burden estimations [18]. The objective of this study was to propose an approach for mapping EQ-5D utilities to existing GBD 2010 and GBD 2013 DWs, and to predict “new” GBD 2010/2013 DWs based on EQ-5D utilities, i.e., for health states currently not included in the GBD studies.

## Methods

### Study design and participants

#### First pilot study

Four health states representing the spread of the GBD 2010/2013 DW distribution were selected (Table 2). In other words, we selected four health states which values shared the GBD 2010/2013 DW distribution in three equal parts. Native speakers translated existing health state descriptions developed by Salomon et al. [1] into French and back-translated them into English (Additional file 1). A written version of the EQ-5D-5 L questionnaire excluding the visual analogue scale part was used and three different versions of the questionnaire were designed. As recommended by Euroquol group, we used the five level EQ-5D questionnaire (EQ-5D-5 L) because the measurement properties of EQ-5D-5 L are superior to the EQ-5D-3 L in terms of feasibility, ceiling effects, discriminatory power and convergent validity [9].

Each version was composed of the same set of health states but with a different order of questioning, to explore possible order effects. The first version presented health states in increasing order of GBD 2010/2013 health state severity, the second version in decreasing order of GBD 2010/2013 health state severity, and the third one did not present health states in a certain order of severity.

The questionnaire consisted of two main parts. The first part was the same in each of the three versions of the questionnaire and included questions about age, sex and marital status. The second part included evaluation of four health states. Participants were asked to imagine being in a certain health state described by the lay descriptions developed by Salomon el al. [3], but without knowing the name of the health state. They would then select the most appropriate level of functioning for each of the five dimensions: mobility, self-care, usual activities, pain/discomfort and anxiety/depression. The respondents were students of the Public Health faculty at the Université catholique de Louvain (UCL), Brussels, Belgium, and were recruited during two courses in June 2014. There was no time limitation to answer the questionnaire and the study leader was present during the study allowing participants to ask any question.

#### Second pilot study

Twenty-seven health states representing the spread of the GBD 2010/2013 DW distribution were selected (Table 2). Native speakers with knowledge of the topic at hand, translated existing health state descriptions developed by Salomon et al. [1] into French and then back-translated them into English (Additional file 1). Dutch translation of the thirty-two health states came for the Haagsma et al. study [2]. A web-based version of the EQ-5D questionnaire excluding the visual analogue scale part, was developed using the Qualtrics software. As for the first study, the first part of the questionnaire included socio-demographics questions, with additional questions on disease/injury experience (yes/no answers), income and educational level. Income categories were derived from Haagsma et al. [2]. The second part included evaluation of four health states. As in the first pilot study, participants were asked to imagine being in a certain health state described by the lay descriptions of Salomon et al. but without knowing the name of the health state [1]. Eight versions of the second part were developed. Each version included evaluation of four health states representing the spread of the GBD 2010/2013 DW distribution. The questionnaire was assigned to participants through a computer algorithm that attributed the questionnaire version with the lowest percentage of participants at the time of assignment. Participants had to answer all questions to validate their participation. The respondents were recruited by email among students of the Public Health faculty at the UCL and among principals of Brussels’s primary and secondary schools and using a social network through general and private messages on Facebook. Snowball sampling [19] was used for the recruitment strategy, i.e., existing study participants were asked to recruit future subjects among their contacts. The data were collected in June 2015. A reminder was sent three weeks after sending the first email to the students of UCL and the principals of Brussels’s schools. Through Facebook, a first request was published on the Facebook public wall of the two main leaders of the study on June 1 2015. Then, three recalls were sent through private messages to all friends of the two main leaders of the study on June 3, 16 and 22, 2015.

### Data analysis

Two steps recommended by the EQ-5D-5 L user guide were followed to transform the EQ-5D-5 L scores reported by each participant into a utility [9]. A function previously developed by the EuroQoL group was used to translate the scores from a 5 L to a 3 L scale, because no Belgian tariffs exist for the 5 L version. The EQ-5D-3 L results were then converted to utilities using the tariff model developed by Cleemput et al. for the Belgian population [10].

### Statistical analysis

For all EQ-5D utilities, overall means, medians, standard deviation and ranges were calculated. Utilities in this study were compared with GBD 2010 and GBD 2013 DWs using Spearman correlation coefficients. We used sample paired t-tests to test whether there was a difference of mean utilities between the pilot studies and the corresponding GBD 2010/2013 DWs.

A locally weighted scatterplot smoothing (loess) regression model was run between the utilities and the logit transformed corresponding GBD 2010/2013 DWs [1, 3]. We arbitrarily chose an anchor point that when DWs equated to 1, utility equated 0 and when DWs equated 0, utility equated 1. Logit transformed DWs were then predicted for each of the utility scores from the loess fit. To retransform the scale and obtain DW values that range from 0 to 1, an inverse logistic transformation was applied to these predicted values.

Analyses were done with R version 3.0.2 and JMP pro 12. R code and developed mapping functions are available through: https://github.com/brechtdv/eq5d-mapping.

## Results

### First pilot study

In total 81 students completed the first survey, 57 were female (70.4%) and 57 had never been married (70.4%). They were on average 29.6 years old (SD 8.4) (Table 1).

**Table 1:** Description of the study population *N* (%) - Mean (SD)

		Study 1 (*n* = 81)	Study 2 (*n* = 393)
		*N* (%)	*N* (%)
Age (years) – Mean (SD)		29.6 (8.4)	36.4 (12.5)
Male		57 (70.4)	104 (26.5)
Marital status	Married	16 (20)	153 (38.9)
	Divorced	7 (8.8)	28 (7.1)
	Widower	0 (0.0)	5 (1.3)
	Separated	0 (0.0)	33 (8.4)
	Never been married	57 (71.3)	174 (44.3)
Income level (in €)	<25 000		70 (17.8)
	25 – 44 000		84 (21.4)
	45 – 52 000		98 (33.4)
	>52 000		57 (14.5)
	Not available		84 (21.4)
Educational level	Primary		1 (0.3)
	Lower secondary		8 (2.0)
	Higher secondary		37 (9.4)
	Bachelor		156 (39.7)
	Master or higher		183 (46.6)
	Not available		2 (0.5)
Disease experience status	Yes		121 (30.8)

Average utilities ranged from 0.667 (SD 0.131) for “Fractures, treated (long term)” to 0.289 (SD 0.203) for “Schizophrenia: acute state” (Table 2).

**Table 2:** Utilities by studies

ID	Health states	Mean Utility study 1	SD Utility study 1	Mean Utility study 2	SD Utility study 2	DW2010	DW2013
1	Fractures, treated (long term)	0.667	0.131	0.694	0.130	0.003	0.005
2	Distance vision, mild			0.826	0.154	0.004	0.003
5	Anemia, mild			0.813	0.138	0.005	0.004
8	Amputation of toe			0.650	0.127	0.008	0.006
11	Asthma, controlled			0.809	0.128	0.009	0.015
22	Claudication			0.644	0.177	0.016	0.014
29	Stroke, long term mild			0.550	0.168	0.021	0.019
37	Disfigurement, level 1 with itch or pain			0.639	0.177	0.029	0.027
47	Fracture of foot bones (long term, without treatment)			0.615	0.151	0.033	0.026
55	Headache tension type	0.530	0.169	0.350	0.204	0.040	0.037
60	Amputation of both legs (long-term with treatment)			0.304	0.244	0.051	0.088
77	Injured nerves (short term)			0.553	0.193	0.065	0.100
96	Fracture tibia, patella (short term)			0.389	0.170	0.087	0.050
107	Musculoskeletal problems (arms moderate)			0.474	0.173	0.114	0.117
111	Severe wasting	0.474	0.204	0.486	0.263	0.127	0.128
149	Crohn’s disease			0.455	0.200	0.225	0.231
155	Parkinson’s disease moderate			0.391	0.201	0.263	0.267
161	Gout, acute			0.193	0.212	0.293	0.295
174	Severe chest injury (short term, with or without treatment)			0.331	0.201	0.352	0.369
186	Fracture of pelvis short term			0.106	0.203	0.390	0.279
193	Headache (migraine)			0.548	0.194	0.433	0.441
200	Rectovaginal fistula			0.449	0.217	0.492	0.501
203	Terminal phase, without medication (for cancers, end-stage kidney/liver disease)			0.317	0.225	0.519	0.569
206	AIDS cases (not received ARV treatment)			0.360	0.228	0.547	0.582
214	Traumatic brain injury (long-term consequences)			0.200	0.257	0.625	0.637
219	Multiple sclerosis (severe)			0.144	0.198	0.707	0.719
220	Schizophrenia, acute	0.289	0.203	0.341	0.192	0.756	0.778

One participant attributed a negative utility (i.e., worse than death) to ‘schizophrenia, acute’ (−0.158) and three participants attributed a utility of one (i.e., perfect health) to ‘severe wasting’.

**Fig. 1:**
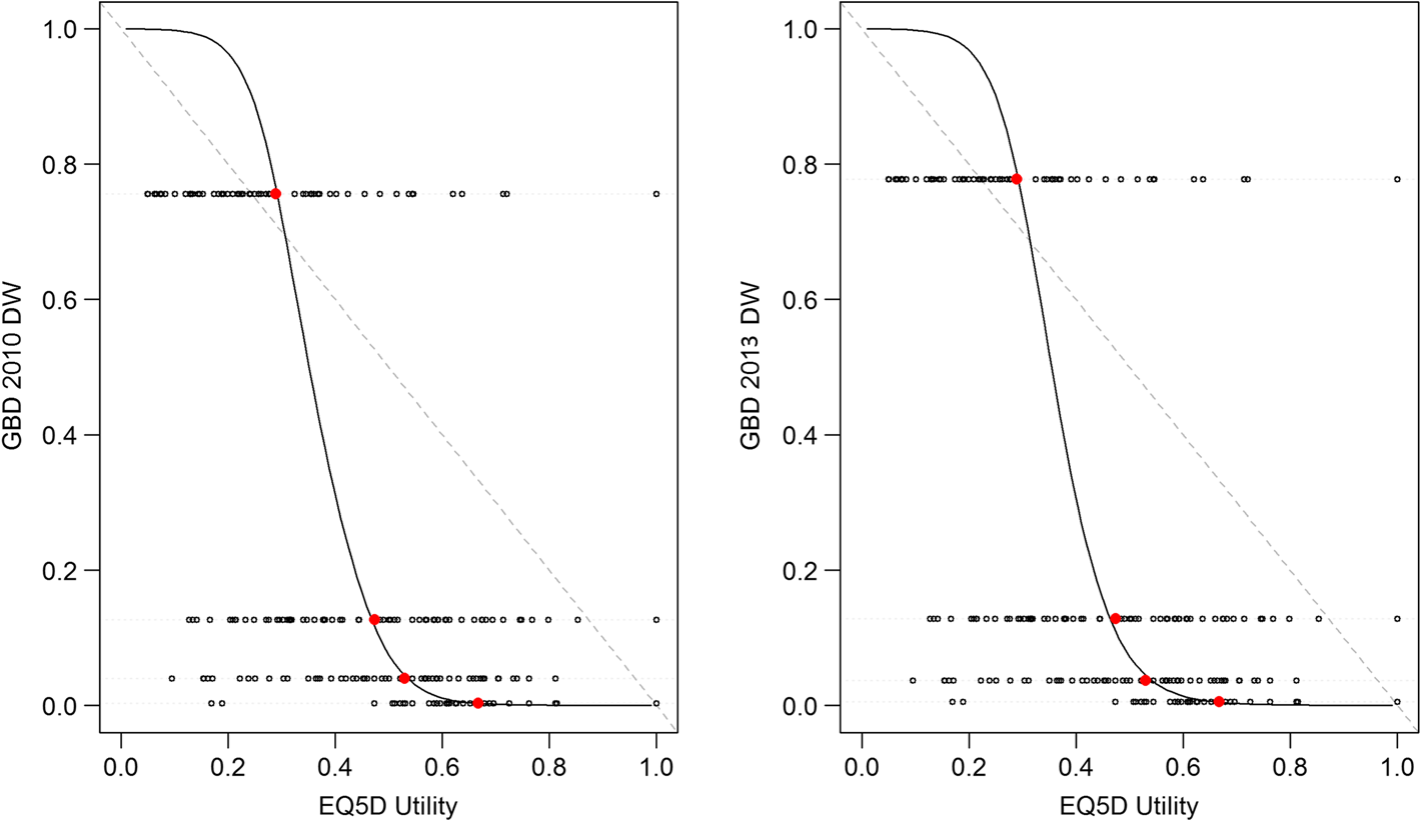
Mapping utilities developed in pilot study 1 vs GBD 2010 DWs and GBD 2013 DWs. Grey dots = individual utility value , red dots = mean utility, grey line = linear regression line, black line = loess regression line

There was a monotonic relationship between derived utilities and predicted GBD 2010/2013 DWs, i.e., the lower the utility, the higher the predicted GBD DW (Fig. 1).

There was a negative correlation between utilities and GBD 2010 DWs (rho = −1, p =0.083) and GBD 2013 DWs (rho = −1, p = 0.083).

There was a significant difference between the complement of the utilities (1 – utility) and GBD 2010 DWs (p = 0.085) and between the complement of the utilities and GBD 2013 DWs (p = 0.099).

‘Fractures treated, long term’ was ranked first, i.e. with the lowest disability weight as in the GBD 2010 study (but ranked third in the GBD 2013 study) and ‘Schizophrenia, acute’ was ranked last, i.e. with the highest disability weight as in the GBD 2010/2013 studies (Table 3).

**Table 3:**
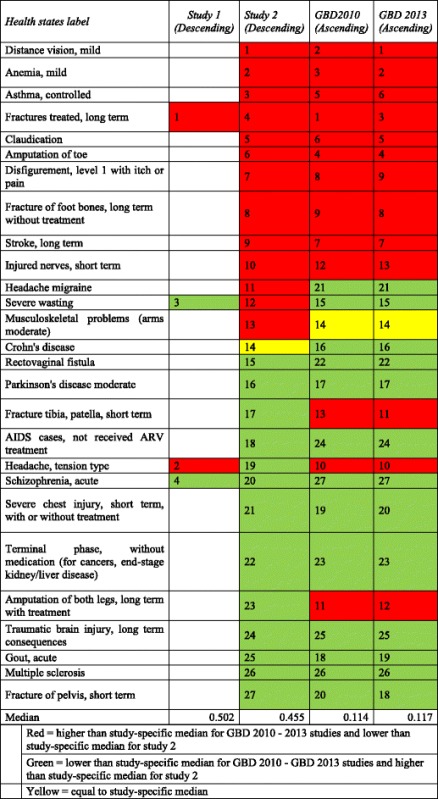
Ranking of the health states by studies

Red = higher than study-specific median for GBD 2010–2013 studies and lower than study-specific median for study 2

Green = lower than study-specific median for GBD 2010 - GBD 2013 studies and higher than study-specific median for study 2

Yellow = equal to study-specific median

### Second pilot study

393 respondents completed the second survey. The average age was 36.4 years (SD 12.5) and 26.3% of the respondents were male. 2.5% were younger than 20, 36% were between 20 and 29 years old, 26% were between 30 and 39 years old and only 4% were older than 60. The majority of the respondents were highly educated, i.e. 39.7% had a bachelor’s and 46.6% had a master’s degree. Most of the respondents had a medium income level, i.e. 54.8% had a household annual income ranging from 25,000 to 52,000 euros (~27,000 US$ - 56,500 US$). Of all respondents, 30.8% already experienced a disease or injury experience in the past and 44.3% had never been married (Table 1).

**Fig. 2:**
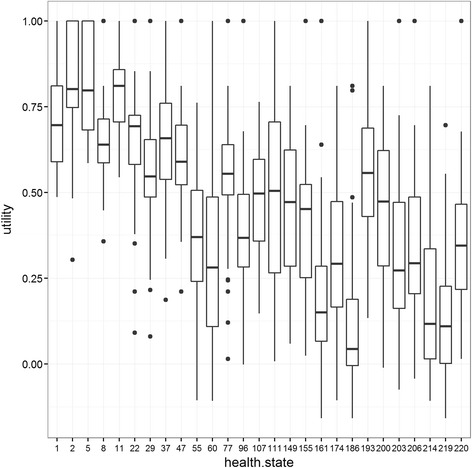
Distribution of utilities derived in pilot study 2. Medians and ranges of the 27 utilities. Black dots represent outliers. Health state IDs are available in Table 2.

The median of the utility distribution of the 27 health states was 0.455 (range: 0.106 - 0.826). The median of the selected 27 GBD 2010 DWs was 0.114 (range: 0.003 – 0.756). The median of the 27 GBD 2013 DW was 0.117 (range: 0.003 – 0.778). According to the utilities, the top three less severe health states in pilot study 2 were ‘Distance vision, mild’, 0.826 (SD 0.154), ‘Anemia, mild’, 0.813 (SD 0.138) and ‘Asthma, controlled’,0.809 (SD 0.128). ‘Fracture of pelvis short term’, 0.106 (SD 0.203), ‘Multiple sclerosis’, 0.144 (SD 0.198) and ‘Gout, acute’, 0.193 (SD 0.212) were the top three more severe health states (Table 2 & 3, Fig. 2). Most of the utilities were between 0.5 and 0.6, and corresponded to the health states with a moderate severity level.

Overall 64 of the participants (16%) in the second pilot study, attributed negative utilities to at least one health state. We obtained negative utilities for 11 health states (41%) among the 27 health states included in the second pilot study (Fig. 2). In health states including negative values, the most common were ‘Fracture of pelvis short term’ (22%), ‘Traumatic brain injury (long-term consequences)’ (20%) and ‘Multiple sclerosis (severe)’ (20%). Conversely, 64 participants (16%) attributed a utility of 1 to at least one health state. The top three most common health states with a utility of 1 were ‘Distance vision, mild’ (25%), ‘Anemia, mild’ (22%) and ‘Asthma, controlled’ (16%).

**Fig. 3:**
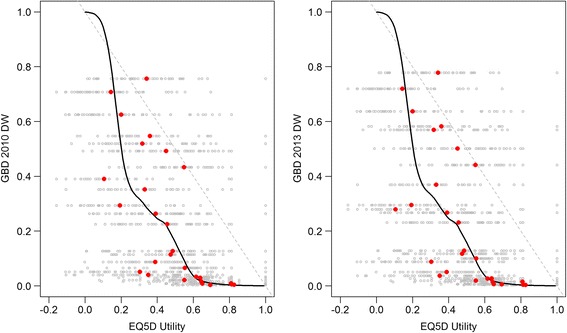
Mapping utilities developed in pilot study 2 versus GBD 2010 DWs and GBD 2013 DWs. Grey dots = individual utility value, red dots = mean utility, grey line = linear regression line, black line = loess regression line

The Spearman rank correlation coefficient between the utilities derived from pilot study 2 and the GBD 2010 DWs equaled −0.808 (p < 0.001). There was a significant difference between the complement of the utilities (1 – utility) and GBD 2010 DWs (p < 0.001). The differences between the complement of the utilities and the GBD 2010 DWs ranged from −0.097 (‘Schizophrenia, acute’) to 0.645 (‘Amputation of both legs (long-term with treatment’). The average difference between 1-utilities and the GBD 2010 DWs was 0.30 (SD 0.18). The three highest observed differences between 1-utilities and the GBD 2010 DWs were ‘Amputation of both legs (long-term with treatment)’ (0.645), ‘Headache tension type’ (0.610) and ‘Fracture tibia, patella (short term)’ (0.524). The three lowest observed differences were noticed for ‘Headache (migraine)’ (0.019), ‘Rectovaginal fistula’ (0.059) and ‘AIDS cases not received ARV treatment)’ (0.093) (Fig. 3).

The Spearman rank correlation coefficient between the derived utilities from pilot study 2 and the GBD 2013 equaled −0.801 (p < 0.001). There was a significant difference between the complement of the utilities (1 – utility) and the GBD 2013 DWs (p < 0.001). The differences ranged from −0.112 (‘Schizophrenia, acute’) and 0.613 (‘Headache tension type’). The average difference between 1-utilities and the GBD 2013 DWs was 0.30 (SD 0.19). The three highest observed differences between 1-utilities and DWs GBD 2013 were for ‘Fracture of pelvis short term’ (0.615), ‘Headache tension type’ (0.613) and ‘Amputation of both legs (long-term with treatment)’ (0.608). The three lowest differences ‘Headache (migraine)’ (0.011), ‘Rectovaginal fistula’ (0.05) and AIDS cases (not received ARV treatment)’ (0.06).

The relationship between the utilities and predicted DWs for both GBD 2010 and 2013 studies was not monotonic (Fig. 3), meaning that both utilities and predicted DWs do not increase/decrease concurrently, and there were major differences in the ranking of health states between the second pilot study and the GBD 2010 and 2013 studies (Table 3). Most of the differences were located in the middle of the distribution, i.e. for health states that were located around the median of the distribution. ‘Amputation of both legs’ was ranked 11^th^ and 12^th^ in the GBD 2010/2013 studies and fell to the 23^rd^ place in this study. ‘Headache tension type’ was ranked 10^th^ in the GBD 2010/2013 studies and 19^th^ in this study. On the other hand, participants of GBD 2010/2013 studies judged more severe ‘Headache migraine’ and ‘Rectovaginal fistula’ which held respectively the 21^st^ and 22^nd^ compared to pilot study 2 where they held the 11^th^ and 15^th^ rank.

### Pilot study 1 versus pilot study 2

We also observed some differences of mean utility between study 1 and study 2. The differences ranged from −0.05 for ‘Schizophrenia, acute’ to 0.180 for ‘Headache tension type’ and we found higher agreement with health states located at the ends of the distribution, i.e. ‘Fractures, treated’ and ‘Schizophrenia’.

For ‘Severe wasting’, most of the participants of the second pilot study assigned a score of three (i.e., moderate) to all five EQ5D dimensions. Participants of the second pilot study evaluated that ‘Severe wasting’ caused more frequently none/moderate (Level 1 & 3) problems for mobility (Dimension 1), no problem of autonomy (D2) and weak (Level 2) pain/discomfort (D4). Participants of the first pilot study evaluated more frequently that ‘Severe wasting’ caused weak (Level 2) problems of mobility (D1), weak (Level 2) problem of autonomy (D2) and none (Level 1) pain/discomfort (D4).

For ‘Headache tension’ the participants of the second study attributed a higher level for three of the five EuroQol dimensions than participants of the first study. Participants of the second study evaluated more frequently that ‘Headache tension’ caused severe pain/discomfort (Level 4 – D4), severe problems to accomplish daily activities (Level 4 – D3) and moderate anxiety/depression (Level 3 – D5). Participants of the first study evaluated more frequently that ‘Headache tension’ caused moderate pain/discomfort (Level 3 – D4), moderate problems to accomplish daily activities (Level 3 – D3) and weak anxiety/depression (Level 2 – D5) (Table 4).

**Table 4:** Dimensions and levels frequency by study

	D1: Mobility		D2: Autonomy		D3: Daily activities		D4: Pain/discomfort		D5: Anxiety/ depression	
	Study 1	Study 2	Study 1	Study 2	Study 1	Study 2	Study 1	Study 2	Study 1	Study 2
ID = 1 Fractures, treated (long term)										
Level 1: None	25%	39%	63%	63%	37%	37%	5%	5%	69%	67%
Level 2: Weak	62%	44%	31%	31%	49%	49%	78%	78%	28%	28%
Level 3: Moderate	11%	17%	6%	6%	12%	12%	15%	15%	1%	6%
Level 4: Severe	2%	0%	0%	0%	1%	1%	1%	1%	1%	0%
Level 5: Extreme/Impossible	0%	0%	0%	0%	0%	0%	1%	1%	0%	0%
Mean	1.91	1.78	1.43	1.33	1.78	1.61	2.16	2.19	1.35	1.39
SD	0.67	0.72	0.61	0.48	0.71	0.73	0.58	0.52	0.57	0.60
ID = 55 Headache tension type										
Level 1: None	52%	30%	53%	48%	6%	2%	1%	0%	19%	16%
Level 2: Weak	37%	24%	30%	14%	32%	10%	12%	6%	47%	18%
Level 3: Moderate	10%	18%	14%	30%	51%	34%	56%	20%	28%	34%
Level 4: Severe	1%	22%	4%	4%	9%	44%	25%	60%	6%	24%
Level 5: Extreme/Impossible	0%	6%	0%	4%	2%	10%	6%	14%	0%	8%
Mean	1.60	2.50	1.68	2.02	2.69	3.50	3.22	3.82	2.22	2.90
SD	0.72	1.30	0.85	1.15	0.82	0.89	0.79	0.75	0.82	1.18
ID = 111 Severe Wasting										
Level 1: None	14%	28%	26%	42%	6%	25%	46%	23%	11%	19%
Level 2: Weak	32%	17%	35%	26%	17%	17%	30%	43%	20%	23%
Level 3: Moderate	27%	28%	27%	23%	43%	26%	20%	28%	37%	30%
Level 4: Severe	27%	19%	12%	6%	30%	25%	5%	6%	27%	23%
Level 5: Extreme/Impossible	0%	8%	0%	4%	4%	8%	0%	0%	5%	6%
Mean	2.68	2.60	2.26	2.04	3.07	2.74	1.84	2.17	2.95	2.74
SD	1.02	1.11	0.98	0.88	0.93	1.10	0.91	0.68	1.06	0.99
ID = 220 Schizophrenia										
Level 1: None	56%	73%	31%	73%	2%	13%	43%	38%	2%	4%
Level 2: Weak	16%	8%	22%	10%	7%	6%	22%	21%	1%	4%
Level 3: Moderate	19%	10%	35%	12%	17%	17%	15%	13%	10%	10%
Level 4: Severe	9%	8%	11%	6%	52%	42%	16%	19%	28%	25%
Level 5 : Extreme/Impossible	1%	2%	1%	0%	21%	21%	4%	8%	58%	58%
Mean	1.84	1.58	2.30	1.50	3.81	3.52	2.15	2.37	4.38	4.29
SD	1.09	1.07	1.07	0.92	0.94	1.28	1.25	1.37	0.90	1.05

Overall, in the first pilot study the scores for mobility (D1) and autonomy (D2) dimensions were in average higher than in the second pilot study. In the second pilot study, the scores for activities (D3), pain/discomfort (D4) and anxiety/depression (D5) dimensions were in average higher than in the first pilot study (Table 4).

## Discussion

DALY-based burden of disease studies play an increasingly important role in public health research [20]. As the required DWs for the health states under study may not be available, flexible and practical ways of eliciting DWs that are comparable with existing GBD DWs are therefore needed. We proposed a mapping approach based on a loess regression model to easily predict any GBD 2010/2013 DW from EQ-5D5L utilities. To our knowledge, the current study is the first to map EQ-5D utilities to GBD 2010/2013 DWs.

Recently, Burstein et al. used loess regression to map SF-12 utilities to GBD 2010 DWs [21]. They showed a weaker rank order correlation (−0.7) between GBD 2010 DWs and SF-12 scores than we found between DWs and utilities (> − 0.8). They also found that the relationship between GBD 2010 DWs and SF-12 scores was non-monotonic. The mapping function developed by Burstein et al. was less satisfactory than in the first pilot study but better than in the second pilot study. Burstein et al. selected 62 health states, collected data from a sample of 3791 respondents and corrected for outliers. Except that participants were enrolled in Seattle and during two GBD workshops, no information was available on age, educational or income level and disease experience status of their study population.

We observed major differences in the validity of the mapping function between both pilot studies, which could be influenced by the different study populations and questionnaire forms. Indeed, a higher prediction quality was observed when we derived utilities from a written version of the EQ-5D questionnaire compared to a web-based questionnaire. One explanation could be that during the first study, which included the written version of the EQ-5D questionnaire, one of the study leaders was present to answer the questions of the participants. This may have increased the understanding of the health state definitions. The wider standard deviations of the second pilot study underscore this hypothesis. Even though we used the updated version of the lay definitions developed by Salomon et al. [3], we still observed that descriptions were not straightforward to understand for lay population. In the second pilot study, we observed a weaker quality of the prediction for more severe disorders, for example ‘Schizophrenia, acute’, ‘Epilepsy severe’ or ‘Rectovaginal fistula’.

In addition, we observed some overlap between the health state descriptions developed by Salomon et al. [3] and the five dimensions included in the EQ-5D questionnaire that could have influenced the health state valuations. For example, the definition of ‘Fracture of pelvis: short term’ was, “…*You have severe pain*, *and cannot walk or do daily activities*” which provided information on the level of mobility, self-care, usual activities and pain/discomfort dimensions included in the EQ-5D5L questionnaire. We observed that utilities for health states with overlap in descriptions had lower variation. For example, in the second pilot study the lowest standard deviation was observed for ‘Amputation of toe’ (SD 0.127) and ‘Asthma, controlled’ (SD 0.128), both of them included information on pain and daily activities in their descriptions.

We also observed that there were some important differences of ranking between GBD 2010/2013 DWs and utilities derived in the second utilities study. Respondents of our study ranked amputation of both legs lower (more severe) and acute schizophrenia higher (less severe) than the respondents of the GBD 2010/2013 studies. In addition to the overlap between the health state descriptions described above, one explanation could be that for participants included in the pilot studies it was more difficult to imagine and evaluate functional limitations (D1 – D3) for psychiatric disabilities than for impaired mobility.

Several limitations may also have influenced the final results.

In the second pilot study, a sample of 27 GBD 2010 health states was used, and each health state was evaluated at least 30 times. Chuang et al. determined that each health state has to be at least evaluated 100 times to be representative of the population of responses [22]. We indeed observed better results in the first study, where each health state was at least evaluated 81 times.

The study population was not representative of the Belgian population. The first study population (*n* = 81) was composed of students in public health, mostly female and young adults. In the second study (*n* = 393), and despite the snowball strategy, most of the participants were between 20 and 39 years (72%), were highly educated. The web-based questionnaire as well as the age of two main coordinators of the study could be an explanation of the study population characteristics. Hopefully, Haagsma et al. demonstrated no significant effects of educational level on DWs for injury consequences in the Dutch population [17]. However other studies, which also used EQ-5D questionnaire, demonstrated that participants aged 18–59 evaluated health states less severely than those aged 60 and over and that older participants attributed less weight to morbidities and pain experience than younger [23, 24].

In addition, some authors reported that the judgment of people who are in a certain health state and health professionals differ significantly from judgment of healthy people [15]. Thirty-one percent of the respondents included in the second study had a disease experience and mostly were expert in public health, which might have influenced the results. Utilities could have been under or over estimated. However we do not recommend to restrict participants to healthy individuals because we believe that utilities have to represent the average ‘preference’ of health of the studied population.

We chose to include negative values of individual utility in the Cleemput’s model, indicating some health states to be worse than death for some participants. We obtained a negative value for one (25%) health state in the first pilot study and for 11 health states (41%) in the second pilot study. For a study including a larger population, it is recommended to constrain values between 0 and 1 to improve the prediction model [25] but in the practice, the issues raised by the negative values for EQ-5D health states are complex [26]. In addition, to perform the final mapping we arbitrarily chose that when DWs equated to 1, utility equated 0, indicating that it exists health state worse than death. This is one of the methodological and philosophical choice difference between the utilities derived from EQ-5D tool and DWs derived from pairwise comparison that could impact the predictions. This is also why assuming the DW to be equal to one minus the utility do not guarantee comparability with the GBD 2010/2013 DWs.

In addition, both ‘short’ and ‘long’ term health states were included in the study and the time framing was not explicitly defined. Some studies demonstrated that the duration of a health state has an impact on the health state valuations and that poor states of health became more intolerable the longer they last [17, 18, 23, 27]. However Salomon et al. showed that the framing of paired comparsion questions in terms of temporary or chronic outcomes in a pairwise comparison did not affect the valuation of the health states [3].

Finally, there are fundamental differences between the pairwise comparison (PC) and EQ-5D techniques.

First, GBD PC was anchored to Population Health Equivalence answers, while EQ5D Flemish tariffs [10] was anchored to Visual Analogue Scale. Although there are systematic differences between those methods, both are indirect and should not affect the mapping. Second, EQ5D questionnaire was designed to assess Quality of Life (QoL) of patients, with a given scenario of health states but DWs were designed to quantify the severity of a single health state. In other words, utilities are patient specific, whereas DWs are health state specific. In this study, we deviate from this original definition by defining health state specific utilities. Both methods also do not evaluate health states on the same dimensions of health [28]. With EQ-5D instrument participants have to evaluate health states on five dimensions of health, each of them including five levels of severity and with pairwise comparison, two health states are presented to healthy people and they have to decide which they regarded as being healthier based on their own judgment and experience. These fundamental differences of methodology can also explain the differences we observed between GBD 2010/2013 DWs and utilities.

## Conclusion

This study suggests the possibility to translate any utility derived from EQ-5D scores into a DW, but also highlights important caveats. We observed a satisfactory result of this methodology when utilities were derived from a population of public health students, a written questionnaire and a small number of health states in the presence of a study leader. The results were however unsatisfactory when utilities were derived from a sample of the general population, using a web-based questionnaire. For the sake of validating the study, we recommend repeating the first study (i.e. using a printed version of the questionnaire coupled with an available support of the study leader and a small number of health states to evaluate) in a larger and more diverse sample to obtain a more representative distribution across educational levels and ages because DWs may vary between and within countries potentially impacting the mapping between utilities and GBD 2010/2013 DWs. Recommendations on future study designs in regards of an optimal sample size are not straightforward to provide, since knowledge on the variability of data in such studies is lacking. Moreover according to Brazier et al., the sample size (number of people giving responses) used in other mapping studies is very broad as it ranged from 68 to 23,647 [29]. We think that further studies using simulation process, could help to answer the sample size. However at least as important as the sample size, is the representativeness of the sample for the population at hand. To avoid selection basis a probability sample (e.g. random sample) of the participants is required.

## Additional file

Additional file 1: Section 1: Survey instrument: Example of one health state Web -questionnaire. (DOCX 30 kb)
