# Supplementary material for: Mapping EQ-5D utilities to GBD 2010 and GBD 2013 disability weights: results of two pilot studies in Belgium
Source: Arch Public Health. 2017 Feb 6;75:6. doi: 10.1186/s13690-017-0174-z (PMC5292789; doi:10.1186/s13690-017-0174-z)
Supplement: Additional file 1: — Section 1: Survey instrument: Example of one health state Web -questionnaire. (DOCX 30 kb) [file 13690_2017_174_MOESM1_ESM.docx]

# APPENDIX

## Section 1: Survey instrument: Example of one health state Web -questionnaire

1. Quel est votre âge (en années)? ....

2. Quel est votre sexe?

- homme
- femme

3. Quel est votre statut marital?

- marié(e)
- divorcé(e)
- veuf/ve
- séparé(e)
- jamais été marié(e)

4. Quel est approximativement le revenu annuel net de votre ménage (toutes ressources confondues)?

- Moins de 7500€
- 7500€ à moins de 15000€
- 15000€ à moins de 22500€
- 22500€ à moins de 30000€
- 30000€ à moins de 37500€
- 37500€ à moins de 45000€
- 45000€ à moins de 52500€
- Plus de 52500€
- Je ne sais pas/ je ne veux pas divulguer l'information

5. Quel est le plus haut niveau d’étude que vous avez achevé jusqu’à présent (école primaire,

école secondaire, bachelier, master, doctorat) ?

- Primaire
- Secondaire inférieur : 3ème année de l'enseignement secondaire
- Secondaire supérieur : 6ème année de l’enseignement secondaire
- Niveau du premier cycle de l’enseignement universitaire (bachelier) ou des formations non universitaires de type court
- Niveau du second cycle universitaire (master) et plus, ou de formations non universitaires de type long
- Je ne sais pas

6. Avez- vous souffert ou souffrez - vous d'une maladie ou des conséquences d'une blessure?

Oui / Non

**QUESTIONNAIRE**

- Imaginez que vous expérimentez l'état de santé suivant:

1. **Vous avez une légère douleur osseuse suite à une fracture dans le passé**

En cochant une seule case dans chacune des cinq catégories ci-dessous, indiquez quelles sont les meilleures descriptions de votre état de santé imaginaire

| Mobilité |  |
| --- | --- |
| Je n’ai aucun problème pour me déplacer à pied | ❑ |
| J’ai des problèmes légers pour me déplacer à pied | ❑ |
| J’ai des problèmes modérés pour me déplacer à pied | ❑ |
| J’ai des problèmes sévères pour me déplacer à pied | ❑ |
| Je suis incapable de me déplacer à pied | ❑ |
| Autonomie de la personne |  |
| Je n’ai aucun problème pour me laver ou m’habiller tout(e) seul(e) | ❑ |
| J’ai des problèmes légers pour me laver ou m’habiller tout(e) seul(e) | ❑ |
| J’ai des problèmes modérés pour me laver ou m’habiller tout(e) seul(e) | ❑ |
| J’ai des problèmes sévères pour me laver ou m’habiller tout(e) seul(e) | ❑ |
| Je suis incapable de me laver ou de m’habiller tout(e) seul(e) | ❑ |
| Activités courantes *(exemples: travail, études, travaux ménagers, activités familiales ou loisirs)* |  |
| Je n’ai aucun problème pour accomplir mes activités courantes | ❑ |
| J’ai des problèmes légers pour accomplir mes activités courantes | ❑ |
| J’ai des problèmes modérés pour accomplir mes activités courantes | ❑ |
| J’ai des problèmes sévères pour accomplir mes activités courantes | ❑ |
| Je suis incapable d’accomplir mes activités courantes | ❑ |
| Douleurs / gêne |  |
| Je n’ai ni douleur ni gêne | ❑ |
| J’ai des douleurs ou une gêne légère(s) | ❑ |
| J’ai des douleurs ou une gêne modérée(s) | ❑ |
| J’ai des douleurs ou une gêne sévère(s) | ❑ |
| J’ai des douleurs ou une gêne extrême(s) | ❑ |
| Anxiété / Dépression |  |
| Je ne suis ni anxieux(se), ni déprimé(e) | ❑ |
| Je suis légèrement anxieux(se) ou déprimé(e) | ❑ |
| Je suis modérément anxieux(se) ou déprimé(e) | ❑ |
| Je suis sévèrement anxieux(se) ou déprimé(e) | ❑ |
| Je suis extrêmement anxieux(se) ou déprimé(e) | ❑ |

## Section 2 : included health states

| **English** | **French** | **Dutch** |
| --- | --- | --- |
| **Fractures: treated, long term** | **Fracture: traitée, à long terme** | **Breuk: behandeld, lange termijn** |
| You have a slight pain in a bone that was broken in the past | Vous avez une légère douleur osseuse suite à une fracture dans le passé | U heeft een lichte pijn in een bot dat in het verleden gebroken is geweest |
| **Distance vision: mild impairment** | **Vision de loin: détérioration modérée** | **Visie: matige verslechtering** |
| You have some difficulty with distance vision, | Vous avez quelques difficultés à voir de loin, par exemple, pour lire des panneaux de signalisation, mais pas d'autres problèmes de la vue | U heeft wat moeite met zicht op afstand, bijvoorbeeld bij het te lezen van verkeersborden, maar geen andere gezichtsproblemen. |
|  |  |  |
| **Amputation of toe** | **Amputation de l'orteil** | **Amputatie van een teen** |
| You have lost one toe, leaving occasional pain and tingling in the stump | Vous avez perdu un orteil, laissant une douleur occasionnelle et des picotements dans le moignon | U heeft een teen verloren, en heeft nu af en toe last van pijn en een tintelend gevoel in de stomp |
| **Asthma controlled** | **Asthme contrôlé** | **Astma behandeld** |
| You have wheezing and cough once a month, which does not cause difficulty with daily activities | Une fois par mois, vous avez une respiration sifflante et de la toux, qui ne provoque aucune difficulté dans vos activités quotidiennes | Een keer per maand, heeft u een piepende ademhaling en hoest die u geen problemen bezorgen in uw dagelijkse activiteiten |
| **Claudication** | **Claudication** | **Kreupelheid** |
| You have cramping pains in the legs after walking a medium distance. The pain goes away after a short rest | Vous avez des crampes dans les jambes après avoir marché une distance moyenne. La douleur s'en va après une courte période de repos | U heeft krampen in de benen na het wandelen van een gemiddelde afstand. De pijn verdwijnt na een korte periode van rust |
| **Stroke: long-term, consequences mild** | **Accident vasculaire cérébral (AVC)** | **Cerebrovasculair accident (CVA)** |
| You have some difficulty in moving around and some weakness in one hand, but are able to walk without help | Vous avez quelques difficultés à bouger et une certaine faiblesse dans une main, mais vous êtes capable de marcher sans aide | U heeft wat moeite met bewegen en enige zwakte in één hand, maar u kan wandelen zonder hulp |
| **Disfigurement: level 1** | **Défiguration: niveau 1** | **Esthetische schade: graad 1** |
| You have a slight, visible physical deformity that others notice, which causes some worry and discomfort | Vous avez une légère déformation physique visible, que les autres personnes remarquent et qui vous cause une certaine inquiétude et de l'inconfort | U heeft een lichte zichtbare fysieke misvorming die andere mensen opmerken en die zorgt voor enige bezorgdheid en ongemak |
| **Fracture of foot bones: long term, without treatment** | **Fracture os du pied: long terme, sans traitement** | **Botbreuk van voet: lang termijn, zonder behandeling** |
| You have a broken foot in the past that did not heal properly. You have pain in the foot and have some difficulty walking | Vous avez eu une fracture du pied dans le passé qui ne s'est pas guéri correctement. Vous avez mal au pied et vous avez quelques difficultés à la marche | U heeft vroeger een voet gebroken en deze is niet correct geheeld. U heeft nu pijn aan de voet en enige hinder bij het wandelen. |
| **Headache: tension-type** | **Maux de tête: type tension** | **Hoofdpijn: type spanning** |
| You have a moderate headache that also affects the neck, which causes difficulty in daily activities | Vous avez un mal de tête modéré qui touche également votre nuque et qui vous cause des difficultés dans vos activités quotidiennes | U heeft een matige hoofdpijn die uitstraalt tot aan de nek en die problemen in uw dagelijkse activiteiten veroorzaakt |
| **Amputation of both legs: long-term, with treatment** | **Amputation des 2 jambes: long terme, avec traitement** | **Amputatie 2 benen met behandeling** |
| You have lost part of both legs, leaving pain and tingling in the stumps. The person has two artificial legs that make moving around possible, with extra effort. | Vous avez perdu une partie des deux jambes, et vous ressentez encore de la douleur et des picotements dans les moignons. Vous avez deux jambes artificielles qui permettent de vous mouvoir avec un effort supplémentaire | U heeft een deel van beide benen verloren, en u voelt nog pijn en een tintelend gevoel in de stompen. U heeft twee comfortabele kunstbenen die u in staat stellen om u voort te bewegen met extra inspanning |
| **Injured nerves: short term** | **Nerf blessé: court terme** | **Zenuwbeschadiging: korte termijn** |
| You have a nerve injury, which causes difficulty moving and some loss of feeling in the affected area | Vous avez une lésion nerveuse qui vous cause des difficultés à mobiliser la zone atteinte et une certaine perte de sensibilité à cet endroit | U heeft een zenuwbeschadiging gehad, die zorgt voor een verminderde beweeglijkheid en een zeker verlies aan gevoeligheid van het aangetaste gebied |
| **Fracture of patella, tibia, fibula or ankle: short term, with or without treatment** | **Fracture de la rotule ou du tibia ou du péroné ou de la cheville** | **Breuk van knieschijf, scheenbeen, fibula of enkel** |
| You have a broken shin bone, which causes severe pain, swelling, and difficulty walking | Vous avez une fracture du tibia qui cause une douleur sévère, un gonflement et des difficultés à marcher | U heeft een fractuur van het scheenbeen, die ernstige pijn veroorzaakt, zwelling en moeite met lopen |
| **Musculoskeletal problems:** | **Problèmes musculaires et squelettiques: Bras, modérée** | **Spier- en skeletproblemen: Armen, matig** |
| You have moderate pain and stiffness in the arms and hands, which causes difficulty lifting, carrying, and holding things, and trouble sleeping because of the pain | Vous souffrez d'une douleur modérée et d'une raideur au niveau des bras et des mains, qui vous causent des difficultés pour soulever, porter, tenir des choses, et qui troublent votre sommeil à cause de la douleur | U lijdt aan een matige pijn en stijfheid in de armen en handen, waardoor u moeite hebt om te tillen, te dragen, en dingen vast te houden, en uw slaap wordt verstoord vanwege de pijn |
| **Severe wasting** | **Perte de poids sévère** | **Zware verlies van gewicht** |
| You are extremely skinny and have no energy | Vous êtes extrêmement maigre et sans énergie | U bent uiterst mager en heeft geen energie |
| **Crohn's disease, moderate** | **Maladie de Crohn modérée** | **Crohn Ziekte, matige** |
| You have cramping abdominal pain, have diarrhea several times a day, and feel very tired for two months every year. When you don't have symptoms, there is anxiety about them returning | Vous souffrez de crampes abdominales, accompagnées des diarrhées plusieurs fois sur la journée. Vous vous sentez très fatigué pendant deux mois chaque année. Quand vous n'avez pas de symptômes, vous êtes anxieux à l'idée que ceux-ci puissent revenir | U heeft buikkrampen, meerdere keren per dag diarree en voelt zich twee maanden per jaar erg moe. Als u geen symptomen hebt, is er angst voor een terugkeer van de symptomen. |
| **Parkinson's disease, moderate** | **Maladie de Parkinson modérée** | **Ziekte van Parkinson** |
| You have moderate tremors and move slowly, wich causes some difficulty in walking and daily activities. You have some trouble swallowing, talking, sleeping, and remembering things | Vous souffrez de tremblements modérés et vous vous mobilisez doucement, ce qui vous cause quelques difficultés à la marche et dans vos activités quotidiennes. Vous avez quelques troubles de la déglutition, de la parole, du sommeil et pour vous souvenir de choses | U heeft matige tremoren en beweegt langzaam, wat zorgt voor wat problemen tijdens het lopen en uitvoeren van dagelijkse activiteiten. U heeft wat moeite met slikken, praten, slapen en het onthouden van dingen |
| **Gout, acute** | **Goute, aiguë** | **Gout, acuut** |
| You have severe pain and swelling in the leg, making it very difficult to get up and down, stand, walk, lift, and carry heavy things. You have trouble sleeping because of the pain | Vous avez une douleur sévère et un gonflement de la jambe, qui provoquent de grosses difficultés à vous lever, vous asseoir, à marcher, à soulever et porter des choses lourdes. Vous avez des troubles du sommeil à cause de la douleur | U heeft ernstige pijn en zwelling van het been, waardoor u grote problemen heeft om op te staan, te zitten, te wandelen, en om zware dingen te tillen en te dragen. U heeft problemen met slapen vanwege de pijn. |
| **Severe chest injury short term, with or without treatment** | **Blessure à la poitrine sévère, court terme, avec ou sans traitement** | **Verwonding aan borstkas, korte termijn, met of zonder behandeling** |
| You have a serious chest injury, which causes severe pain, shortness of breath and anxiety | Vous avez une sérieuse blessure à la poitrine, qui vous cause des douleurs sévères, le souffle court lorsque vous respirez et de l'anxiété | U heeft een ernstige verwonding  aan de borstkas, die leidt tot erge pijn, kortademigheid en angst. |
| **Fracture of pelvis: short term** | **Fracture du pelvis: court terme** | **Pelvis Breuk** |
| You have a broken pelvis bone, with swelling and bruising. You have severe pain, and cannot walk or do daily activities | Vous avez une fracture du petit bassin, avec un gonflement et une ecchymose (contusion, bleu). Vous souffrez d'une douleur sévère, vous ne savez pas marcher ou réaliser les tâches de la vie quotidienne | U heeft een gebroken bekken met zwelling en kneuzingen. U heeft erg pijn, u kunt niet wandelen en u heeft problemen met het uitvoeren van dagelijkse activiteiten |
| **Headache** | **Mal de tête: migraine** | **Hoofdpijn** |
| You have severe, throbbing head pain and nausea that cause great difficulty in daily activities and sometimes confine you to bed. Moving around, light, and noise make it worse | Vous souffrez d'une douleur pulsatile à la tête et de nausées qui vous causent de grosses difficultés dans les activités de la vie quotidienne et qui peuvent parfois vous obliger à rester au lit. Se déplacer, la lumière ou le bruit peuvent aggraver les symptômes | U heeft een ernstige, kloppende hoofdpijn en misselijkheid die ertoe leiden dat u veel moeite heeft met dagelijkse activiteiten en die soms vereisen dat u in bed blijft. Rondbewegen, licht of geluid kunnen de symptomen verergeren. |
| **Rectovaginal fistula** | **Fistule recto-vaginale** | **Recto-vaginaal fistul** |
| You have a abnormal opening between her vagina and rectum causing flatulence and feces to escape through the vagina. You get infections in your vagina, and have pain when urinating | Vous avez une communication anormale entre le vagin et le rectum, qui cause des flatulences et des selles qui s'échappent par le vagin. Vous souffrez d'infections vaginales et des douleurs lorsque vous urinez | U heeft een abnormale opening tussen de vagina en rectum, waardoor winderigheid en ontlasting ontsnappen via de vagina. U heeft last van vaginale infecties en pijn bij het plassen. |
| **Terminal phase, without medication** | **Phase terminale, sans médication** | **Terminale fase, zonder behandeling** |
| you have lost a lot of weight and have constant pain. You have no appetite, feels nauseous, and needs to spend most of the day in bed | Vous avez perdu beaucoup de poids et vous souffrez d'une douleur constante. Vous n'avez pas d'appétit, vous vous sentez nauséeux, et vous passez la plupart de votre journée temps dans votre lit | U heeft veel gewicht verloren en doorlopend pijn. U heeft geen eetlust, u voelt zich misselijk, en moet het grootste deel van de dag in bed doorbrengen. |
| **AIDS case: not recieving antiretroviral treatment** | **SIDA: Sans Traitement anti-rétroviral** | **AIDS: zonder anti-retroviraal behandeling** |
| You have severe weight loss, weakness, tiredness, cough and fever, and frequent infections, skin rashes and diarrhea | Vous avez perdu beaucoup de poids, vous êtes faible, fatigué, vous toussez et avez de la fièvre, vous souffrez régulièrement d'infections, vous avez des éruptions cutanées et des diarrhées | U verloor veel gewicht, u bent zwak, u bent moe, u hoest en u hebt koorts. U heeft regelmatig last van infecties, u heeft huiduitslag en diarree |
| **Traumatic brain injury long-term consequences severe, with or without treatment** | **Vous avez des lésions cérébrales traumatiques** | **U heeft traumatische hersenletsels gehad** |
| You cannot think clearly and have frequent headaches, memory problems, difficulty concentrating and dizziness. You are often anxious and moody, and you depend on others for feeding, toileting, dressing and walking | Vos idées ne sont pas claires, vous avez des maux de tête fréquents, des problèmes de mémoire, des difficultés de concentration et des vertiges. Vous êtes souvent anxieux, d'humeur changeante, et vous dépendez des autres pour vous nourrir, pour vous laver, vous habiller, et marcher | U kunt niet helder nadenken, u heeft vaak hoofdpijn, problemen met het geheugen, moeite met concentratie en duizeligheid. U bent vaak angstig, humeurig en, u bent afhankelijk van anderen om u te voeden, te wassen, te kleden om te lopen |
| **Multiple sclerosis severe** | **Sclérose en plaques sévère** | **Multiple sclerose** |
| You have slurred speach and difficulty swallowing. You have weak arms and hands, very limited and stiff leg movement, you have loss of vision in both eyes and cannot control urinating | Vous avez des troubles de l'élocution et de déglutition. Vous souffrez de faiblesse dans les bras et les mains, vous avez des mouvements très limités au niveau des jambes et celles - ci sont raides. Vous avez perdu la vue des deux yeux et vous avez une incontinence urinaire | U heeft spraak- en slikproblemen. U heeft zwakke armen en handen, erg beperkte en stijve beweging van de benen, verlies van gezichtsvermogen aan beide ogen en u heeft urineverlies |
| **Schizophrenia: acute state** | **Schizophrénie sévère** | **Ernstige schizofrenie** |
| You hear and see things that are not real and are afraid, confused, and sometimes violent. You have great difficulty with communication and daily activities, and sometimes want to harm or kill yourself | Vous entendez et voyez des choses irréelles et vous êtes effrayé, confus, parfois violent. Vous avez un gros problème de communication et pour les activités de la vie quotidienne. Parfois vous voulez vous faire du mal ou vous suicider | U hoort en ziet dingen die er niet zijn en u bent bang, verward, soms gewelddadig. U heeft ernstige problemen met communicatie en dagelijkse activiteiten. Soms wilt u zich pijn doen of zelfmoord plegen |
| **Anaemia: mild** | **Anémie: légère** | **Bloedarmoede: lichte** |
| You feel slightly tired and weak at times, but this does not interfere with normal daily activities | Vous vous sentez légèrement fatigué et faible par moment, mais ceci n'interfère pas dans vos activités la vie quotidienne | U voelt zich soms een beetje moe en zwak, maar dit heeft geen invloed op uw dagelijkse activiteiten. |
